# Salivary level of microRNA-146a and microRNA-155 biomarkers in patients with oral lichen planus versus oral squamous cell carcinoma

**DOI:** 10.1186/s12903-023-03155-z

**Published:** 2023-06-29

**Authors:** Masoumeh Mehdipour, Minoo Shahidi, Fahimeh Anbari, Homa Mirzaei, Soudeh Jafari, Azam Kholghi, Ehsan Lotfi, Soheila Manifar, Fatemeh Mashhadiabbas

**Affiliations:** 1 Oral Medicine Department, School of Dentistry, Shahid Beheshti University of Medical Sciences, Daneshjoo Blvd, Evin, Chamran high way, Tehran, 1983963113 Iran; 2 Hematology and blood banking Department, Faculty of Allied Medicine, Iran University of Medical Sciences, Tehran, Iran; 3 Department of Medical Biotechnology, Faculty of Allied Medical Science, Iran University of Medical Sciences, Tehran, Iran; 4 Oral Medicine Department, School of Dentistry, Tehran University of Medical Sciences, Tehran, Iran; 5 Oral Pathology Department, School of Dentistry, Shahid Beheshti University of Medical Sciences, Tehran, Iran

**Keywords:** Lichen Planus, oral, Squamous cell carcinoma of head and neck, MicroRNAs, Saliva

## Abstract

**Background:**

Oral lichen planus (OLP) is a chronic inflammatory disease of the oral mucosa, which has potential for malignant transformation. MicroRNAs play an important role in immunopathogenesis of OLP, and may be used for prediction of its malignant transformation. This study aimed to assess the salivary level of microRNA-146a and microRNA-155 biomarkers in patients with OLP and oral squamous cell carcinoma (OSCC).

**Methods:**

In this case-control study, unstimulated saliva samples were collected from 60 patients, including 15 patients with dysplastic OLP, 15 OLP patients without dysplasia, 15 patients with OSCC, and 15 healthy controls according to the Navazesh technique. After RNA extraction, the expression of microRNA-146a and microRNA-155 was quantified by real-time quantitative polymerase chain reaction (RT-qPCR). The data were analyzed by the Kruskal-Wallis and Dunn-Bonferroni tests.

**Results:**

The difference in expression of microRNA-146a and microRNA-155 among the four groups was significant (P < 0.05). Pairwise comparisons of the groups showed significantly higher expression of microRNA-146a in OLP (P = 0.004) and dysplastic OLP (P = 0.046) patients compared with the control group. Up-regulation of this biomarker in OSCC patients was not significant compared with the control group (P = 0.076). Up-regulation of micro-RNA-155 was only significant in OLP group, compared with the control group (P = 0.009). No other significant differences were found (P > 0.05).

**Conclusion:**

Considering the altered expression of MicroRNA-146a and microRNA-155 in dysplastic OLP and OSCC, their altered expression may serve as an alarming sign of malignancy. However, further investigations are still required.

## Introduction

Oral lichen planus (OLP) is a chronic inflammatory disease of the oral mucosa, which has different clinical manifestations. OLP is more common in middle-aged women and has a prevalence of 0.1–2.2% in the general population. Its potential for malignant transformation is reportedly 1–2% annually [1]. There are some controversies about malignant transformation risk factors in patient OLP, some of these risk factors are tobacco, alcohol, clinical form, or intraoral localizations of OLP [1].

Although the role of cell-mediated immunity through the T-lymphocytes in development of OLP has been previously confirmed, the exact etiology of OLP has not yet been clearly understood. Evidence shows that stress, genetics, and infections may play a role in development of OLP [2].

OLP has a wide range of clinical manifestations, and its diagnosis based on the clinical criteria is possible. However, histopathological diagnosis is imperative for the atrophic and erosive types, due to their potential for malignant transformation [3]. Controversy exists regarding the malignant transformation potential of OLP. However, at present, OLP is generally considered as a lesion with potential for malignant transformation [2].

Evidence shows that micro ribonucleic acids (microRNAs) play an important role in immunopathogenesis of OLP, and may be used for prediction of its malignant transformation. MicroRNAs play a pivotal role in regulation of the immune function and prevention of autoimmune diseases. They are single-stranded non-coded 18 to 25-nucleotide small molecules that regulate post-transcriptional gene expression by targeting the 3-untranslated area of the messenger RNAs (mRNAs) to down-regulate or stop the process of transcription. Gene regulation mediated by microRNAs is critical for the normal cell functions such as cell mitosis, differentiation, and apoptosis such that in one-third of human mRNAs, post-transcriptional gene expression is controlled by epigenetics [4–6].

It has been shown that CD8 + cytotoxic T cells play a role in development of OLP by degeneration of oral epithelial basal layer cells [7]. Also, immune and inflammatory factors and cytokines such as interferon-gamma and tumor necrosis factor-alpha play important roles in development of OLP. MicroRNAs have a close correlation with cytokines in different inflammatory conditions. Some changes in the expression of microRNAs are associated with chronic inflammatory processes and malignant transformation in oral precancerous conditions [6].

Considering the over-expression of microRNA-146a and microRNA-155 in cancerous lesions, they may be able to serve as efficient biomarkers for assessment of possible carcinogenesis. MicroRNA-155 is encoded by the MIR-155 gene, which is localized to the human chromosome band 21q21.3. This miRNA is processed from an exon of the non-coding RNA known as BIC [4]. MicroRNA-155 has numerous applications, and is closely correlated with inflammation and immune system regulation. According to some studies, microRNA-155 is a strong biomarker related to OLP [4, 8]. Altered expression of microRNA-155 is believed to have a significant effect on the biological properties of lymphocytes [9]. Rather et al. evaluated tissue specimens of oral squamous cell carcinoma (OSCC) patients and showed that oncogenic activity of micro-RNA-155 decreased the activity of tumor suppressor CDC73 and increased the risk of OSCC as such [10]. MicroRNA-146a, as an inhibitor of Th1-cell differentiation in human T cells acting via targeting the protein kinase C epsilon (PRKCε), which is part of a functional complex consisting of STAT4 and PRKCε, controls Th1-cell differentiation in human CD4 + T lymphocytes [11]. Also, recent evidence showed that micro-RNA-146a had an important role in inflammatory diseases and regulation of T-lymphocytes [11]. Similarly, some other studies showed over-expression of microRNA-146a in tissue samples of OSCC patients [12, 13]. This over-expression was also noted in metastatic lesions [14]. Huffaker et al. indicated the involvement of microRNA-146a and mciroRNA-155 in regulation of the immune response [15]. These two microRNAs appear to have opposite effects on inflammation since microRNA-155 induces T-lymphocytes, while mciroRNA-146a inhibits T-lymphocytes; they have mutual effects as well [15]. However, some others reported no correlation between them [16]. In OLP patients, in particular, the expression of microRNA-146a and microRNA-155 increases in peripheral blood mononuclear cells [5, 8].

Saliva samples can be used for measurement of biomarkers since saliva reflects the body status and can be used for diagnostic purposes [17]. Arantes et al. demonstrated that microRNAs can be used as diagnostic biomarkers for detection of cancer due to their specific expression in some tumors, and stability in tissues, blood circulation, and body fluids [18]. Saliva can serve as a source of molecular and microbial information and may reveal the presence of a disease condition [18]. Use of salivary biomarkers has been suggested as a reliable non-invasive modality for early detection of OSCC, and can serve as a suitable alternative to invasive biopsy techniques, which is currently the only way for diagnosis of oral cancer in primary stages.

Considering the increasing prevalence of risk factors related to oral cancer [19], and the rising rate of cancer-related morbidity and mortality in Iran, cancer prevention and control is currently a priority, and primary screening of high-risk populations is imperative [20]. Concerning the close relationship of microRNA-155 and microRNA-146a with the immune system, and the variations in the immune system function in different races [21], this study aimed to assess the salivary level of microRNA-146a and microRNA-155 biomarkers in patients with OLP and OSCC.

## Materials and methods

This case-control study was conducted on 15 OLP patients with dysplasia, 15 OLP patients without dysplasia, 15 patients with OSCC, and 15 healthy controls. The study protocol was approved by the ethics committee of Shahid Beheshti University of Medical Sciences (IR.SBMU.DRC.REC.1399.044).

The sample size was calculated to be 15 in each group according to a previous study [22] assuming the effect size of 0.45, alpha = 5%, and power of 80% using ANOVA fixed effect model of G Power. Thirty patients with clinical manifestations of OLP were selected and underwent incisional biopsy to assess the trend of microscopic cellular changes in them. Fifteen OSCC patients were also selected among those presenting to the Oral Medicine Departments of School of Dentistry, Shahid Beheshti University of Medical Sciences and Cancer Institute of Imam Khomeini Hospital after excisional biopsy and histopathological confirmation. Fifteen age- and sex-matched healthy controls with no history of oral lesions or systemic diseases were also included. Finally, due to the coronavirus disease 2019 pandemic, 15 non-dysplastic OLP, 8 dysplastic OLP, and 4 OSCC cases from the study by Mehdipour et al. [22] were also included in this study after gaining permission. The samples had been stored at -80 °C in RNAlater storage solution (Sigma Aldrich, Merck, Germany) [23]. The remaining samples were collected according to the criteria mentioned in a similar previous study [22].

Gender, age, type and manifestations of lesion, location of lesion, duration of involvement, and the existing signs and symptoms were all recorded.

After ensuring no involvement of the skin and genital mucosa and no other malignancy, autoimmune diseases, immunodeficiency, hepatitis, HIV infection, or cardiovascular disease, no smoking and alcohol consumption, and no other oral lesion or periodontitis, written informed consent was obtained from all patients to use their information and biopsy samples for research purposes. A total of 60 patients, including 15 OLP patients with histopathological diagnosis of dysplasia (with different grades), 15 patients with histopathologically confirmed OLP without dysplasia, 15 patients with histopathologically confirmed OSCC, and 15 healthy controls were enrolled. In order to check the presence or absence of dysplasia, patients with OLP were subjected to incisional biopsy from the erosive, ulcerative, and plaque-like areas in which dysplasia is more likely than other areas [22]. The incisional biopsy samples of OLP patients were independently evaluated by two oral and maxillofacial pathologists, and those results suggestive of OLP, and early invasive SCC were excluded.

### Saliva collection

Unstimulated saliva samples were collected from the participants between 8 a.m. to 13 p.m. by the Navazesh technique [24]. According to this technique, the patients were requested to refrain from eating, drinking, and oral hygiene measures (toothbrushing or use of mouthwash) for at least 1 h prior to saliva sampling. The participants were requested to rinse their mouths and then spit into a 15-mL RNase-free Falcon tube in a seated position within 30 min until 3–5 ml of saliva was collected. Next, 1 mL of RNAlater (Sigma Aldrich, Merck, Germany) was added to the samples and they were stored at -80 °C until RNA extraction [22].

### RNA extraction

After removing from the freezer, the saliva samples were centrifuged at 2000 rpm at 4 °C for 20 min to remove debris and floating cells. The transparent supernatant was collected by a 1000-landa (µL) sampler and transferred into a 1.5 µL tube; 1 mL of RNX (Trizol; Anacell Teb, Tehran, Iran) was added to it and pipetted by a sampler. The microtubes were vortexed for 5–10 s and kept at room temperature for 5 min; 200 µL of chloroform was added, manually mixed for 15 s, and placed on ice for 10 min. Next, they were centrifuged at 12,000 rpm at -4 °C. The aqueous phase was removed by a sampler and transferred into a new RNase and DNase-free microtube. Isopropanol was added in an amount equal to the aqueous phase and gently mixed manually. To enhance the deposition of sediment, it was refrigerated at -20 °C overnight. Next, it was centrifuged again at 12,000 rpm at -4 °C. The supernatant was discarded, 1 mL of 75% ethanol was added to the microtube and shortly vortexed. It was then centrifuged at 7500 rpm at -4 °C for 8 min. The supernatant was discarded until the microtube was completely dried. Next, DEPC was dissolved in 20 µL of distilled water. Thermo Scientific NanoDrop One was used to ensure the purity of RNA. The RNA ratios (260/280) were reported in nanograms per microliter (ng/µL).

### Complementary DNA (cDNA) synthesis from RNA

The cDNA required for real-time quantitative polymerase chain reaction (RT-qPCR) was synthesized from the extracted RNA using a cDNA synthesis kit (Anacell Teb, Tehran, Iran) according to the manufacturer’s instructions. All steps of the procedure were performed on ice under a laminar hood in sterile conditions. A specific primer designed by the stem-loop technique was used for this purpose. To prepare the RNA stock solution for cDNA synthesis, the RNA was defrosted and the followings were mixed: 0.5 µL RT primer, 1.125 µL RNA, 0.125 µL dNTP Mix (10 mM each), 0.125 µL RNase Inhibitor (20 U/µl), 0.125 µL ExcelRT™ Reverse Transcriptase (200 U/µl), and 0.5 µL RT Buffer 5X, yielding a total volume of 2.5 µL. In the next step, 2 µL of the stock solution was added to 0.2 RNase and DNase-free Microtubes, and 0.5 µL primers of each gene added to it. The microtube containing the template, primer, enzyme and buffer was placed in a thermocycler (PEQ Star) for cDNA synthesis. The thermal protocol was as follows: 30 min at 16 °C, 30 min at 42 °C, and 5 min at 85 °C.

### RT-qPCR

The synthesized cDNA was mixed with SYBER Green master mix (Anacell Teb) and specific primers for each microRNA in 0.1 µL microtube in a metal rack. U6 gene was used as the internal control and housekeeping gene. RT-qPCR was performed as duplicate for each sample with one cycle of denaturation and 40 cycles of amplification using real-time PCR device (Roche, Switzerland) according to the manufacturer’s instructions. The thermal protocol for PCR was as follows:

Hold: 15 min at 95 °C to activate DNA Tag polymerase.

Cycling: 15 s at 95 °C for DNA denaturation.

Annealing: 60 s at 60 °C for annealing.

### Statistical analysis

Data were analyzed using SPSS version 25 (IBM Co., Armonk, NY, USA). The frequency, percentage, mean, standard deviation, median, and interquartile range were reported in the descriptive data. Since the data were found to have non-normal distribution, the Kruskal-Wallis test was used to compare the four groups regarding the expression of microRNA-146a and microRNA-155. The Dunn-Bonferroni test was applied for pairwise comparisons of the groups. P < 0.05 was considered statistically significant.

## Results

Thirty OLP patients were evaluated. One patient was excluded from the dysplastic OLP group due to saliva sample inadequacy. Of 15 non-dysplastic OLP patients, 11 were females and 4 were males. Of 15 dysplastic OLP patients, 10 were females and 5 were males. Of 15 OSCC patients, 9 were males and 6 were females.

The mean age was 40 ± 14 years in the control group, 52 ± 16 years in non-dysplastic OLP group, 54 ± 12 years in the dysplastic OLP group, and 50 ± 14 years in the OSCC group.

Location of the lesion was widely variable in the oral cavity of patients.

**Fig. 1:**
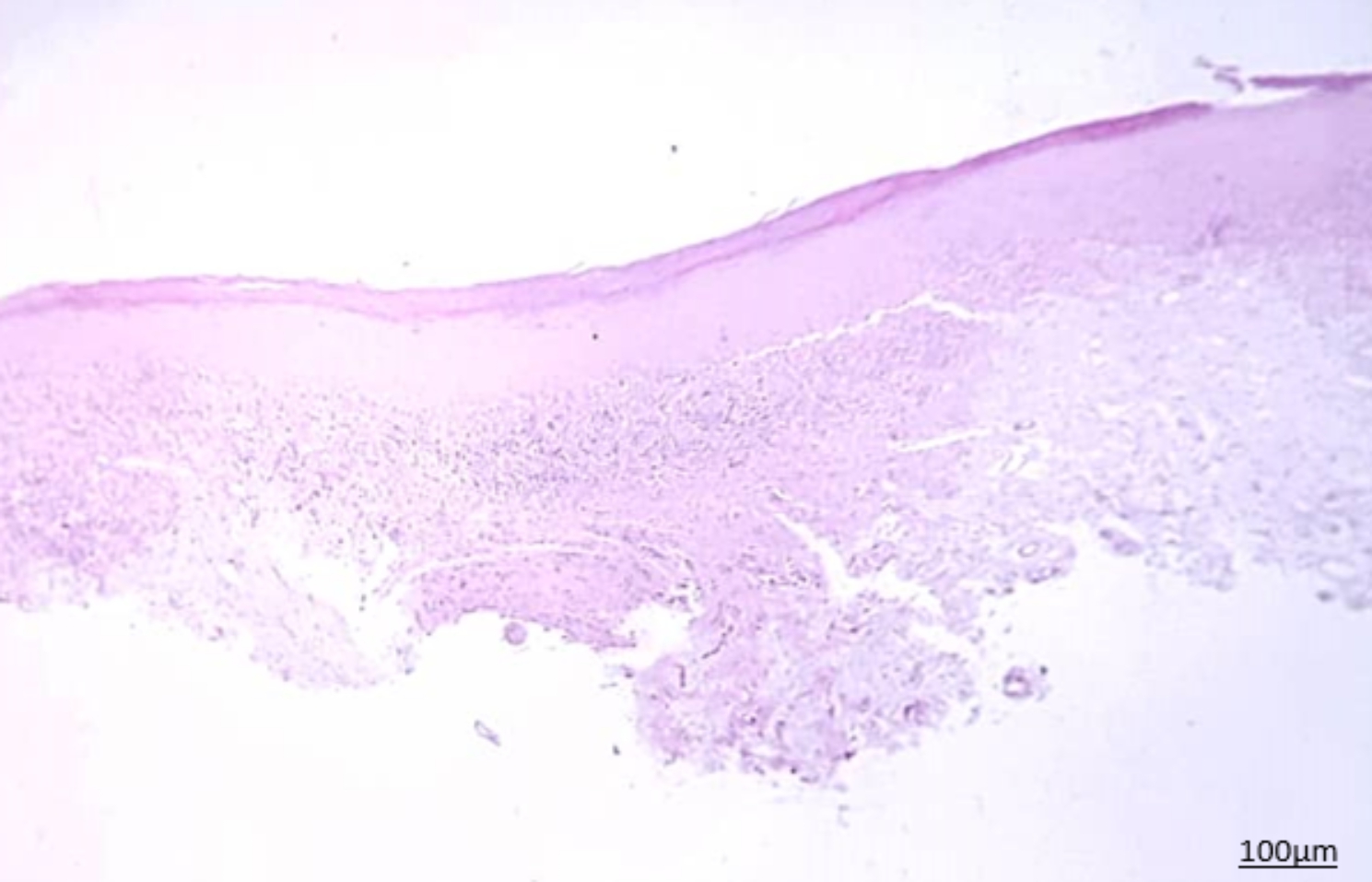
Histopathological micrograph of non-dysplastic OLP (x100 magnification)

**Fig. 2:**
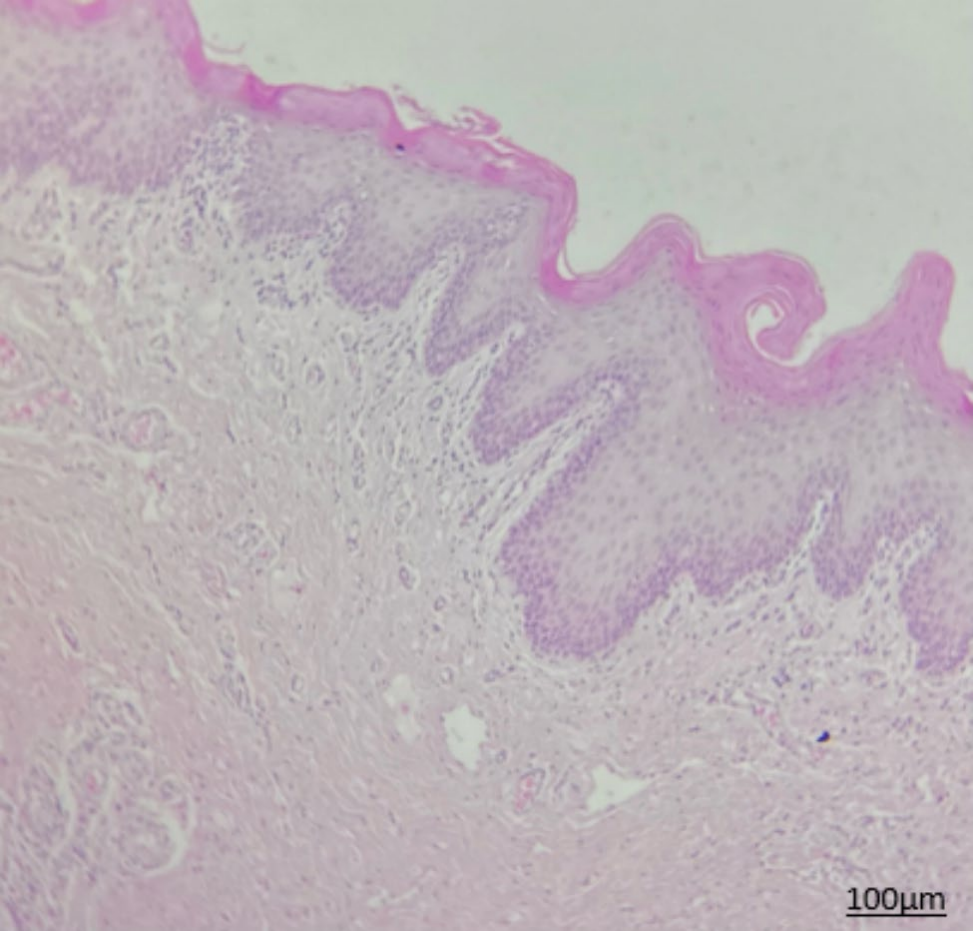
Histopathological micrograph of OLP with mild dysplasia (x100 magnification)

**Fig. 3:**
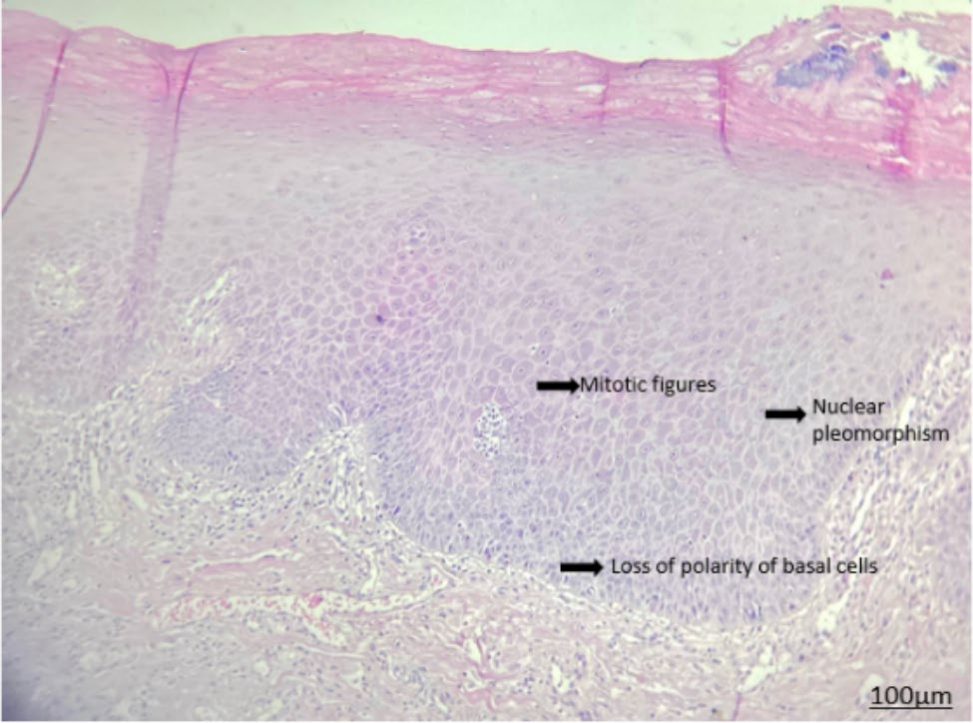
Histopathological micrograph of OLP with moderate dysplasia (x100 magnification)

**Fig. 4:**
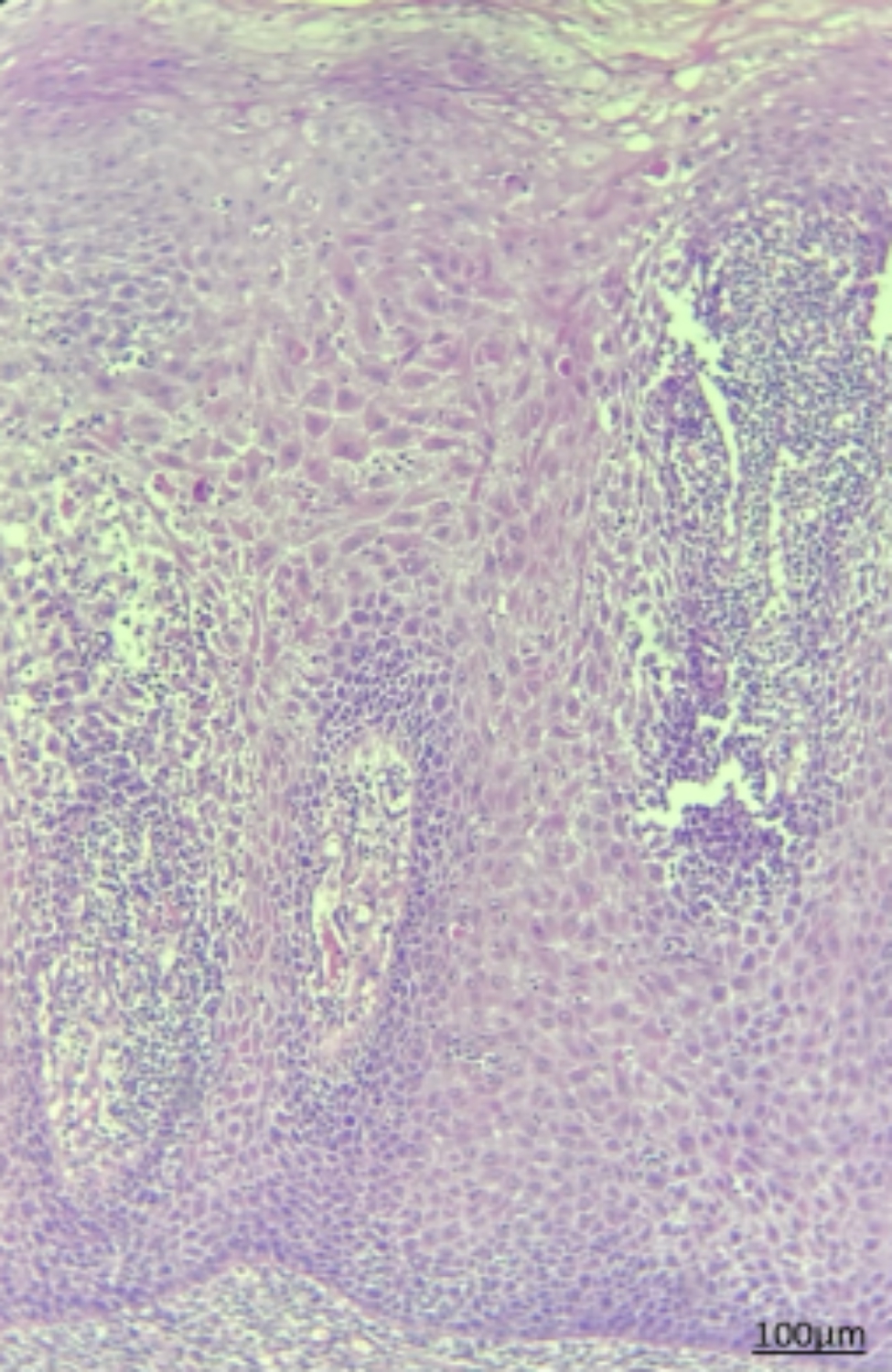
Histopathological micrograph of OLP with severe dysplasia (x100 magnification)

**Fig. 5:**
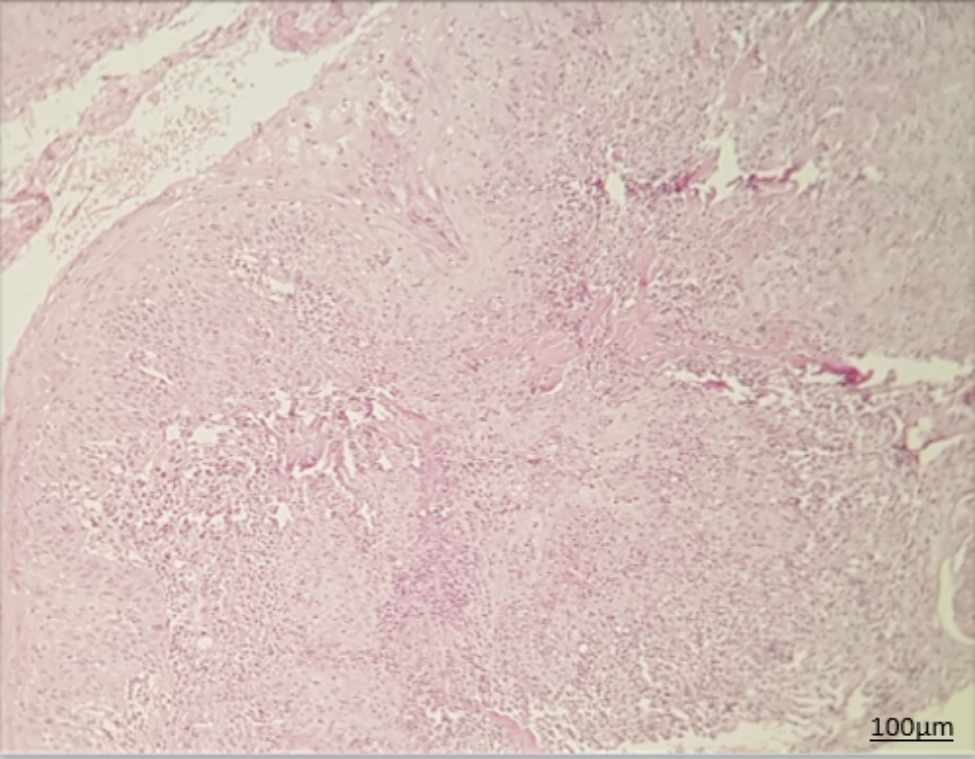
Histopathological micrograph of OSCC (x100 magnification)

Histopathological assessment of biopsy specimens and their re-evaluation by another pathologist showed no evidence of malignancy in 15 patients with non-dysplastic OLP (Fig. 1). In 15 dysplastic OLP patients, dysplastic changes were noted; out of which, 7 had mild dysplasia (42.85%, Figs. 2), 4 had mild to moderate dysplasia (28.57%), 3 had moderate dysplasia (21.42%, Fig. 3), and 1 had severe dysplasia (7.14%, Fig. 4). The cases with lichenoid dysplasia were excluded. Figure 5 shows histopathological micrograph of an OSCC patient.

### MicroRNA-146a

**Fig. 6:**
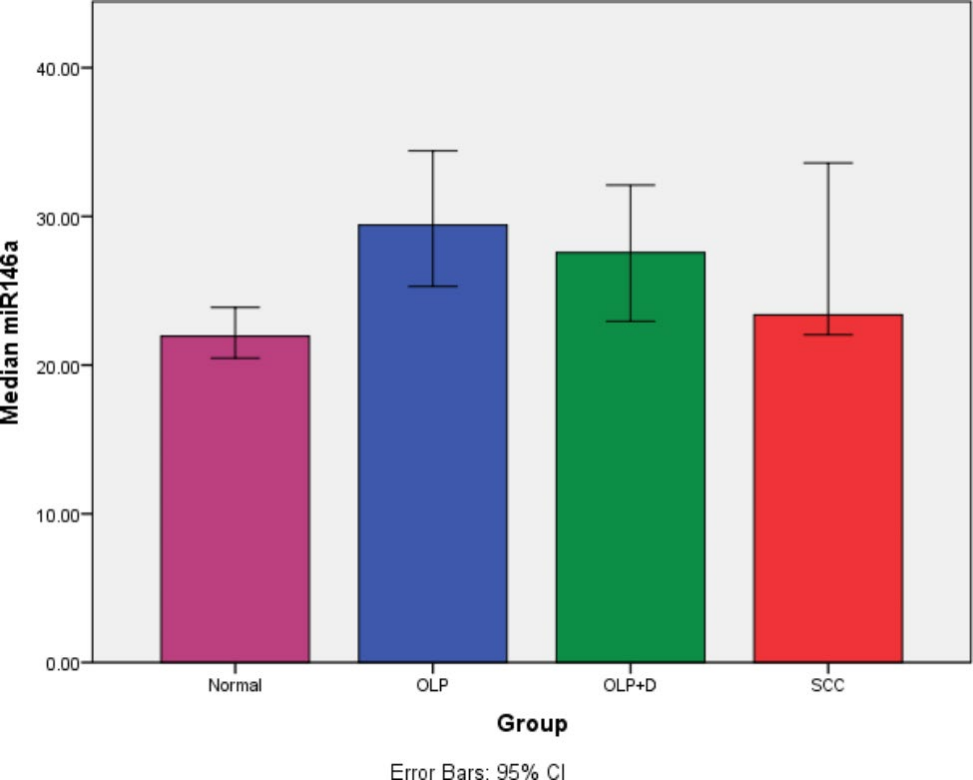
The average expression level of microRNA-146a in different groups with a 95% confidence interval

The Kruskal-Wallis test showed a significant difference in expression of microRNA-146a among the four groups (P = 0.013). Pairwise comparisons by the Dunn-Bonferroni test showed minimum expression of microRNA-146a in healthy controls. The maximum expression of this biomarker was noted in non-dysplastic OLP group with a significant difference with healthy controls (P = 0.004). Also, the expression of this biomarker was significantly higher in dysplastic OLP compared with the control group (P = 0.046). No other significant differences were noted (P > 0.05, Table 1; Fig. 6).

**Table 1:** Pairwise comparisons of the groups regarding the expression of microRNA-146a

Group (I)	Group (J)	Mean difference	P value
Healthy control	OLP	-7.485	0.004*
	Dysplastic OLP	-5.62	0.046*
	SCC	-1.44	0.076
OLP	Dysplastic OLP	1.865	0.329
	SCC	6.045	0.064
Dysplastic OLP	SCC	4.18	0.295

*Statistically significant

### MicroRNA-155

**Fig. 7:**
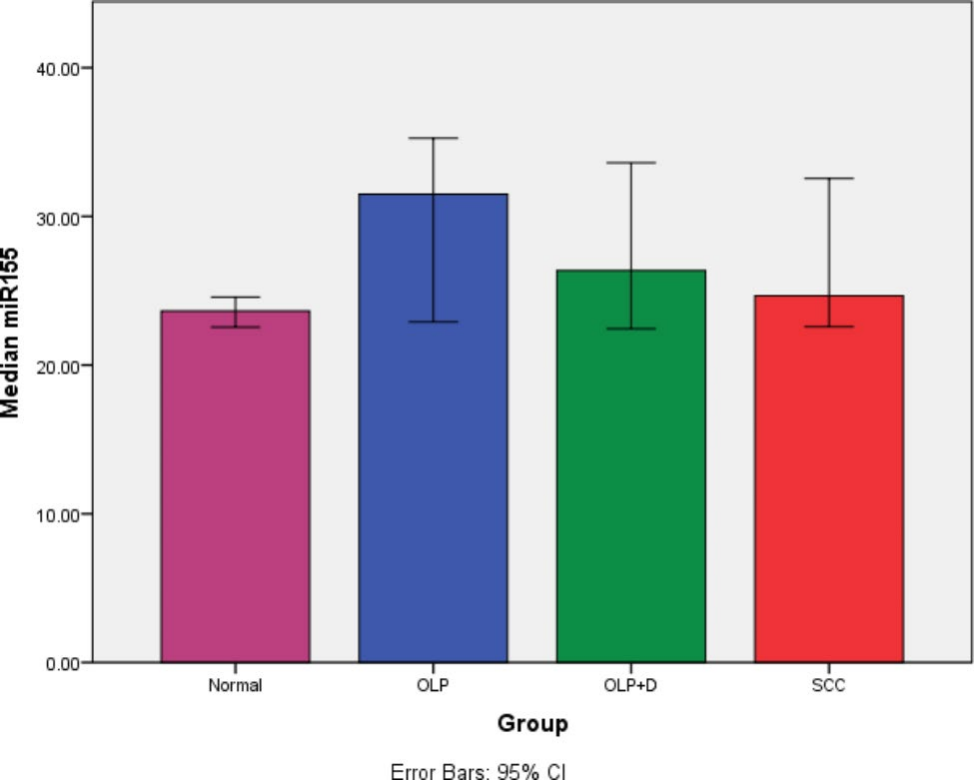
The average expression level of microRNA-155 in different groups with a 95% confidence interval

The Kruskal-Wallis test showed a significant difference in expression of microRNA-155 among the four groups (P = 0.045). Pairwise comparisons by the Dunn-Bonferroni test showed minimum expression of microRNA-155 in healthy controls. Maximum expression of this biomarker was noted in OLP patients with a significant difference compared with healthy controls (P = 0.009). No other significant differences were noted (Table 2; Fig. 7).

Assessment of the expression of microRNAs in males and females and in those under and over 40 years of age revealed no significant difference (P > 0.05).

**Table 2:** Pairwise comparisons of the groups regarding the expression of microRNA-155

Group (I)	Group (J)	Mean difference	P value
Healthy control	OLP	-7.78	0.009*
	Dysplastic OLP	-2.735	0.134
	SCC	-1.03	0.151
OLP	Dysplastic OLP	5.135	0.259
	SCC	6.84	0.095
Dysplastic OLP	SCC	1.705	0.566

*Statistically significant

## Discussion

This study assessed the salivary level of microRNA-146a and microRNA-155 biomarkers in patients with OLP and OSCC. The results showed that the difference in expression of microRNA-146a and microRNA-155 among the four groups was significant (P < 0.05). Pairwise comparisons of the groups showed significantly higher expression of microRNA-146a in OLP (P = 0.004) and dysplastic OLP (P = 0.046) patients compared with the control group. Up-regulation of this biomarker in OSCC patients was not significant compared with the control group (P = 0.076). Up-regulation of micro-RNA-155 was only significant in OLP group, compared with the control group (P = 0.009). No other significant differences were found (P > 0.05). Expression of microRNA had no significant correlation with age or gender. A similar previous study found no significant correlation between age and gender and expression of microRNAs either [25].

Altered expression of microRNAs in the serum, blood, plasma, and saliva samples of patients with head and neck cancer as potential biomarkers with minimal invasion for early detection or determination of prognosis of patients has recently gained the spotlight [26]. Recent studies have focused on the role of microRNAs in the pathogenesis and severity of OLP [8, 27]. However, they mainly assessed the tissue and serum samples of OLP patients [16].

The accuracy and feasibility of the assessment of microRNAs in the saliva of OSCC and OLP patients have been previously documented [28]. Thus, the expression of microRNA-155 and microRNA-146a in the saliva samples of OLP and OSCC patients was evaluated in the present study.

Over-expression of microRNA-146a in OLP patients indicates the involvement of this microRNA in pathogenesis of OLP. The present results were in line with previous findings that showed over-expression of microRNA-146a in OLP patients compared with healthy controls [4]. Also, altered expression of this biomarker has been reported in other inflammatory and autoimmune diseases [29–31]. Liang et al. evaluated the expression of microRNA-155, microRNA-146a, and microRNA-146b in peripheral blood mononuclear cells and tissue specimens of OLP patients and healthy controls [27]. They also analyzed the correlation of microRNA expression and clinical features of OLP. The results of RT-PCR showed that the expression of microRNA-155 in peripheral blood mononuclear cells of OLP patients was significantly higher than that in controls. Also, the expression of microRNA-146a in OLP patients was significantly higher than that in controls, which was in line with the present results. However, in contrast to the present findings, they found no significant difference in expression of microRNA-146a in peripheral blood mononuclear cells between the OLP and control groups. This difference may be due to the assessment of extracellular compared with intracellular microRNAs. A recent study used Western blotting and RT-qPCR and showed elevated expression of microRNA-146a in Treg cells, and increased proliferation and apoptosis of cells in OLP [32]. These results obtained by assessment of tissue specimens were in agreement with the present results obtained by assessment of saliva samples of patients. This similarity may be explained by the role of microRNA-146a in the process of inflammation and T-cell-mediated immunity in OLP [11].

Liu et al. evaluated the expression of microRNA-146a in peripheral blood CD4 + T-cells and local OLP lesions, and its association with clinical presentation of OLP using RT-PCR [33]. They found no significant difference in expression of microRNA-146a by the peripheral blood CD4 + T-cells between the OLP and control groups, which was different from the present findings. This difference may be due to the fact that they analyzed serum samples while we assessed the saliva samples.

Several studies have shown alterations in expression of microRNA-155 in OLP patients. Similar to the present findings, Liu et al. [33] reported an increase in expression of microRNA-155 in peripheral blood mononuclear cells and OLP lesions in OLP patients compared with healthy controls. Moreover, they demonstrated that the expression of microRNA-155 was significantly correlated with disease severity. Nonetheless, in-depth studies on the mechanisms of involvement of microRNA-155 in OLP are limited. As mentioned earlier, immune factors play important roles in pathogenesis of OLP. MicroRNA-155 is closely correlated with the immune system regulation [8], which may explain its over-expression in OLP, as indicated in the present study. Also, microRNA-155 is the first microRNA that was recognized as an oncogene [34]. Its over-expression has been reported in different cancer types such as the breast cancer, colon cancer, cervical cancer, and lung cancer. This particular microRNA is pivotal for tumor development, and mainly serves as a tumorigenic factor [35].

Lerner et al. evaluated blood samples of patients with head and neck cancer and found no significant difference in expression of microRNA-155 and microRNA-146a between the cancer patients and healthy controls [26]. Based on Yan et al. study that revealed similarities in the distributions of the saliva and plasma proteomes by Gene ontology analysis [36], we can conclude that these results in the blood that were proven in the Lerner et al. study [26] can be agreed with the results of our study in the saliva. They concluded that considering the role of biomarkers in proliferation and migration of tumor cells, expression of these biomarkers may be used as an indicator of prognosis of head and neck cancer.

Expression of microRNA-155 in OSCC has been previously investigated. Gombos et al., and Emami et al. found over-expression of this biomarker in tissue and serum samples of OSCC patients [37, 38]. Meng Wu et al. revealed that ARID2 is a target gene of microRNA-155-5p and the loss of ARID2 expression in OSCC patients significantly correlated with poor survival [39]. Lin et al. stated that microRNA-146a expression in swabbed collectives enables the differentiation between normal mucosa and OPMD/OSCC, independent of their histopathological severity [40]. The present results also revealed over-expression of microRNA-155 in OSCC patients compared with healthy controls; however, this increase was not significant. Variations in the results may be due to the assessment of microRNA molecules extracellularly and in the saliva compared with their intracellular assessment in tissue and blood cells.

Relatively small sample size due to COVID-19 pandemic was a limitation of this study. To overcome this limitation, we had to use the samples from the study by Mehdipour et al., [22] and thus, we could not change the eligibility criteria, which was another limitation of this study. A larger sample size may yield more significant results. Further studies with larger sample size are required to confirm the present results. Also, future studies may evaluate both saliva and serum samples of patients to obtain more reliable results and can focus on the correlation between the biomarkers and histological findings.

## Conclusion

Considering the altered expression of microRNA-146a and microRNA-155 in dysplastic OLP and OSCC, their altered expression may be used as an alarming sign of malignancy. However, further investigations are still required.

## Data Availability

The datasets used and/or analyzed during the current study are available from the corresponding author on reasonable request.

## List of abbreviations

microRNAs: Micro ribonucleic acids
OLP: Oral lichen planus
OSCC: Oral squamous cell carcinoma
RT-qPCR: Real-time quantitative polymerase chain reaction
